# Biohacking the human gut microbiome for precision health and therapeutic innovation

**DOI:** 10.3389/fmicb.2026.1776983

**Published:** 2026-03-24

**Authors:** Jhommara Bautista, Andrés López-Cortés

**Affiliations:** Cancer Research Group (CRG), Faculty of Medicine, Universidad de Las Américas, Quito, Ecuador

**Keywords:** biohacking, diet-based modulation, inflammation, microbial therapeutics, synthetic-biology approaches, the human gut microbiome, the human-gut-brain axis

## Abstract

Biohacking, the self-directed application of biotechnology, digital tools, and lifestyle interventions, has rapidly converged with gut microbiome science to create adaptive, individualized, and minimally invasive precision-health paradigms. This narrative review integrates current evidence on diet-based modulation, microbial therapeutics (probiotics, prebiotics, postbiotics, and fecal microbiota transplantation), and synthetic-biology approaches (engineered strains and phage or synthetic consortia) within a multi-omics and continuous-phenotyping framework. Mechanistically, short-chain fatty acids (SCFAs), bile-acid derivatives, and tryptophan catabolites operate as endocrine-like mediators linking gut microbial ecology with host immunity, metabolism, and neuroendocrine signaling. Pathways mediated by microbial metabolites underpin translational applications that span metabolic optimization, through improved insulin sensitivity, reduced adiposity, and attenuation of inflammation, and neurocognitive enhancement via the microbiome-gut-brain axis. Evidence from oncology further indicates that microbial metabolites and engineered taxa remodel stromal and immune niches, shaping therapeutic response and disease progression. Concurrently, emerging digital infrastructures, wearables, biosensors, metabolic avatars, and AI-driven “health twins,” enable real-time, closed-loop modulation of host-microbe dynamics. Persistent challenges include methodological heterogeneity, safety concerns regarding live biotherapeutics and unsupervised fecal microbiota transplantation (FMT), fragmented regulation, and vulnerabilities in cyberbiosecurity and data equity. We propose a translational roadmap emphasizing standardized metadata (STORMS), validated reference frameworks, longitudinal multi-omics for causal inference, strain-level safety genomics, and governance integrating ethical and cybersecurity oversight. Under these conditions, microbiome-focused biohacking may evolve from anecdotal experimentation into a more reproducible and scientifically grounded component of preventive and personalized medicine. This manuscript is presented as a narrative and conceptual review, integrating validated microbiome research with emerging biohacking frameworks while explicitly distinguishing evidence-based findings from exploratory or speculative concepts.

## Introduction

The convergence of biohacking and the human gut microbiome research is increasingly shaping new paradigms for precision health, enabling individuals to intervene in their biological systems through data-driven, self-directed strategies. Biohacking, broadly defined as the do-it-yourself (DIY) use of biotechnology, digital tools, and lifestyle interventions to optimize human performance and health, has expanded beyond niche communities into mainstream health innovation. Historically rooted in self-experimentation and transhumanist ideals, this movement increasingly incorporates the microbiome as a central axis for physiological modulation, disease prevention, and performance enhancement. DIY biology communities and biohackers employ diverse methods, from dietary modulation and probiotic regimens to circadian alignment, engineered microbial consortia, and digital self-tracking platforms, to recalibrate microbiome-host interactions in ways that aim to maximize resilience and optimize health outcome (Gaspar et al., 2019; Gangadharbatla, 2020; Lindfors, 2024).

The human gut microbiome represents a dynamic ecosystem with a critical role in metabolism, immune regulation, and neuroendocrine signaling. Its plasticity makes it particularly attractive for precision biohacking interventions that leverage individualized, non-invasive, and adaptive strategies. Technological advances in wearable sensors, digital phenotyping, and embedded technologies are accelerating this shift toward “quantified self” models, in which microbial signatures are tracked and optimized alongside physiological parameters such as sleep, heart rate, cognitive performance, and dietary intake (Gangadharbatla, 2020). Embedded or wearable biosensors capable of monitoring microbial metabolites, inflammatory biomarkers, or gut-brain signaling molecules offer continuous feedback loops that can inform behavioral or therapeutic adjustments in real time. Data-driven personalization closely reflects the core principles of precision health, emphasizing disease prevention and early detection through high-resolution individual monitoring (Gambhir et al., 2018).

Culturally and ideologically, contemporary biohacking blends technoscientific rationalism with biomimetic imaginaries and alternative medicine logics, framing nature and the body as programmable systems to be optimized rather than passively maintained. Interventions frequently emulate or amplify “natural” regulatory processes, such as circadian rhythms, microbiota-gut-brain communication, or host immune modulation (Bautista et al., 2025g; Nazir et al., 2025). This biomimetic orientation is seen in interventions ranging from fasting protocols and thermal therapies to targeted supplementation and engineered microbial formulations. By integrating these into self-directed protocols, biohackers position the microbiome as both target and mediator of personalized health enhancement (Lindfors, 2024).

A particularly influential trajectory in this space involves age modulation and longevity biohacking, which views the microbiome as a leverage point for delaying or reversing biological aging processes. Through strategies such as dietary fiber enrichment, short-chain fatty acids (SCFAs) modulation, intermittent fasting, and probiotic/prebiotic cycling, biohackers aim to enhance metabolic flexibility, reduce inflammaging, and maintain immune competence over time. It aligns with broader transhumanist perspectives that regard ageing as a modifiable biological condition rather than an unavoidable process of decline (Sato et al., 2025; Cozza et al., 2022).

Beyond philosophical framings, the motivations and frameworks of biohacking reveal a structured but heterogeneous landscape. As documented in recent qualitative and conceptual analyses, biohacking practices can be classified into strands such as DIYbio experimentation, wellness and health optimization, spiritual-augmentative practices, and technological futurism (Lorrimar, 2025). Within this framework, the microbiome is increasingly recognized as a central target of intervention, a programmable interface leveraged to enhance cognition, optimize metabolism, improve stress resilience, and achieve therapeutic benefits (Bautista et al., 2025f; Bui, 2024).

Grassroots biohacking practices often mirror, and in some instances precede, advances in clinical precision health. This emerging field integrates multi-omics profiling, continuous physiological monitoring, and predictive analytics to enable early disease detection and personalized prevention strategies, with the exposome, particularly the composition and activity of the microbiome, occupying a pivotal role in these frameworks (Bautista et al., 2025d; Porcari et al., 2025). By capturing the dynamic interaction between host genetics, microbial ecology, and environmental inputs, biohacking practices can complement formal precision health frameworks, potentially contributing to distributed health monitoring networks (Gambhir et al., 2018).

Nutritional modulation represents a cornerstone of microbiome-targeted biohacking. Diet is one of the most potent modulators of gut microbial diversity and metabolic output. Personalized nutrition strategies based on microbial profiling enable tailored interventions that go beyond the one-size-fits-all dietary guidelines. For example, individualized manipulation of microbiota-accessible carbohydrates, dietary fibers, and polyphenols can enhance the production of beneficial metabolites such as SCFAs, modulate inflammatory signaling, and influence energy balance (Song and Shin, 2022; Arshad et al., 2025).

However, this increasing accessibility also exposes novel risks. As shown in security studies, the democratization of biological tools introduces potential biosecurity vulnerabilities, including malicious manipulation of DNA sequences or synthetic microbial strains (Islam et al., 2019). Similarly, the regulatory frameworks surrounding genetic biohacking remain limited, with concerns regarding safety, efficacy, consent, and public health impact still insufficiently addressed (Zettler et al., 2019). Biohacking technologies possess a dual-use character, requiring governance strategies that balance the advancement of innovation with the need to prevent potential misuse.

**Figure 1:**
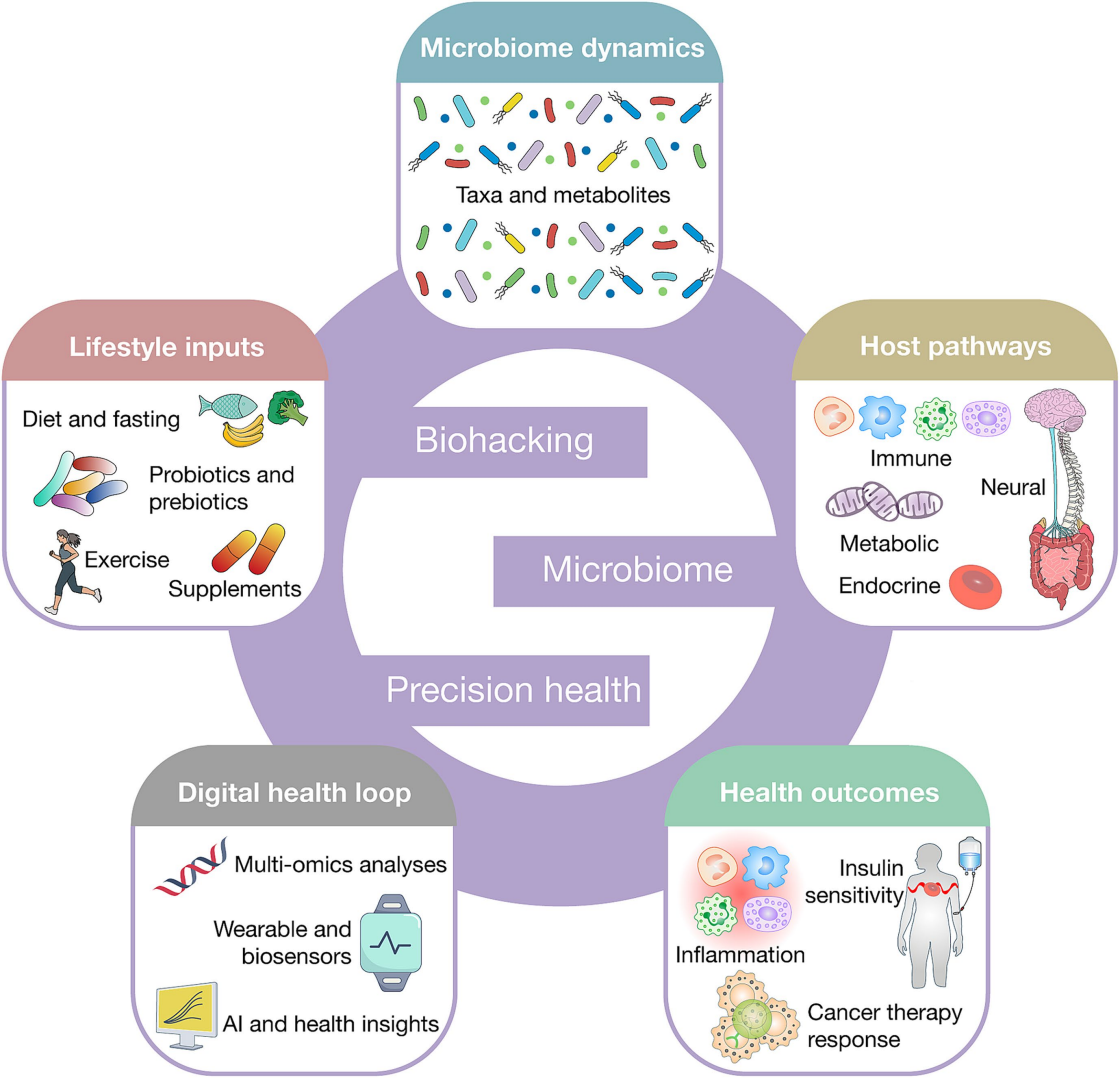
Biohacking-microbiome-precision health closed-loop model. This figure depicts a closed-loop framework in which lifestyle and biohacking interventions modulate microbiome composition and function, shaping host immune, metabolic, neuroendocrine, and epigenetic pathways. Microbiome-derived metabolites translate these interactions into measurable health outcomes, including metabolic control, inflammation, cognition, and therapeutic response. Continuous monitoring through wearables, multi-omics, and AI-driven analytics feeds back to personalize and refine interventions, enabling adaptive precision health.

Emerging digital infrastructures, such as metabolic avatars and AI-driven health twins, are amplifying the personalization and scalability of these interventions. Digital biohacking platforms model individual metabolic and microbial dynamics to generate adaptive dietary and lifestyle recommendations, effectively merging real-time data acquisition with predictive modeling (Abeltino et al., 2024). Such approaches have the potential to optimize caloric intake, reshape microbial ecology, and minimize the environmental footprint of dietary habits, thereby linking personal health optimization with broader goals of planetary sustainability. These interconnected components are summarized in a closed-loop biohacking-microbiome framework for precision health, linking lifestyle inputs to microbial functions, host biological pathways, and digitally enabled feedback for iterative personalization (Figure 1).

Accordingly, this review adopts a narrative and integrative approach rather than a systematic or clinical meta-analytic framework. Its aim is to synthesize established microbiome science with emerging biohacking practices, while critically contextualizing speculative technologies within current biological, translational, and regulatory boundaries.

### Modulation strategies in biohacking integrating diet, microbial modulation and microbial engineering

Biohacking strategies integrating dietary modulation, microbial manipulation, and microbial engineering are increasingly explored as potential tools to modulate host physiology and optimize health outcomes through targeted microbiome interventions. At the foundation of these strategies, diet functions as a primary and dynamic modulator of the gut ecosystem, shaping microbial composition, functional diversity, and metabolic outputs. Macronutrient composition, particularly fiber and resistant starches, has been shown to alter microbial biomass and SCFA production, influencing host energy balance, enteroendocrine signaling, and systemic inflammation. Personalized nutritional interventions tailored to an individual’s baseline microbiota can enhance precision in modulating metabolic pathways, improve gut barrier function, and optimize immune homeostasis, providing a cornerstone for microbiome-centered biohacking protocols (Song and Shin, 2022; Corbin et al., 2023).

Dietary biohacking strategies are increasingly enhanced by digital and algorithmic platforms that integrate real-time metabolic and behavioral data, enabling personalized dietary interventions aimed at both health optimization and environmental sustainability. For instance, digital biohacking approaches leveraging metabolic avatars allow fine-tuning of caloric intake and macronutrient distribution, resulting in effective weight management and reduced metabolic risk (Abeltino et al., 2024). Individualized dietary modulation can swiftly alter microbial diversity and function, illustrating how targeted feeding patterns serve as precision tools to direct microbiome-host interactions toward health-promoting outcomes.

Beyond diet, microbial modulation through biotic strategies, including probiotics, prebiotics, postbiotics, and FMT, represents the second pillar of biohacking interventions. Probiotic and prebiotic formulations can restore eubiosis, modulate immune responses, and enhance production of key metabolites such as butyrate and propionate, which are crucial for epithelial integrity and immune tolerance (Ji et al., 2023). FMT, as a “whole microbiome transfer,” has demonstrated clinical efficacy in specific indications and shows potential to restore colonization resistance, recalibrate immune tone, and modulate systemic pathways relevant to metabolic, inflammatory, and even oncologic diseases. Its expanding application, from recurrent infection to chronic inflammatory and metabolic disorders, illustrates the translational potential of engineered microbiota reshaping as a biohacking tool (Yadegar et al., 2024; Baydoun et al., 2025).

While much of the current evidence remains preclinical, advances in synthetic biology have enabled the rational design of probiotic strains with programmable functionalities. The third strategic layer involves microbial engineering, leveraging synthetic biology to design probiotic strains with programmable functionalities. Engineered bacteria can be tailored to deliver therapeutic molecules, modulate signaling pathways, or act as biosensors in real time (Ma et al., 2022). CRISPR-based editing and chassis optimization have enabled the creation of bacterial strains with enhanced colonization capacity, targeted immune modulation, and metabolic rewiring. Notably, these engineered probiotics are now being explored as delivery vehicles for mucosal immunization and metabolic regulation (Huang et al., 2024; Rutter et al., 2022). In parallel, phage therapy and synthetic consortia further expand the biohacking toolkit, enabling precision targeting of pathogenic taxa while preserving beneficial microbial networks (Airola et al., 2023).

These three axes, diet, microbial modulation, and engineering do not act in isolation but form a synergistic network that can be optimized to reprogram microbiome-host interactions in a controlled and personalized manner. Diet creates the ecological landscape; microbial modulation adjusts community structure; and microbial engineering introduces targeted functionalities, enabling an unprecedented degree of control over host physiology. Such integration aligns with the next frontier of precision health, where personalized, multi-layered microbiome modulation becomes a programmable therapeutic interface. As such strategies advance, rigorous clinical validation and standardized protocols will be crucial to transform biohacking from experimental innovation into scalable, evidence-based health interventions (Song and Shin, 2022; Airola et al., 2023; Rutter et al., 2022).

Recent insights from oncology microbiome research reveal that microbial modulation exerts systemic effects extending beyond metabolic regulation to include immune and stromal remodeling. Evidence from metastatic models demonstrates that gut and tumor-associated microbiota shape host physiology through metabolite-mediated signaling, influencing angiogenesis, immune tolerance, and epithelial-mesenchymal dynamics (Bautista et al., 2025e; Lei et al., 2025). Findings indicate that microbiome-targeted biohacking interventions, including engineered probiotics and metabolite-driven dietary modulation, may be harnessed to reprogram host-microbe communication networks governing inflammation, regeneration, and longevity. Complementary metabolomic profiling further supports that microbial metabolites, including bile acid derivatives, indoles, and polyamines, orchestrate host energy balance and stress adaptation through receptor-mediated and epigenetic pathways (Bautista and López-Cortés, 2025b; Hitch et al., 2022).

### Personalization and multi-omics integration for precision biohacking

The era of precision biohacking leverages the convergence of multi-omics technologies, artificial intelligence, and microbiome analytics to personalize interventions that optimize human physiology, cognition, and longevity. Integrating data from genomics, transcriptomics, proteomics, metabolomics, and metagenomics allows for increasingly high-resolution characterization of individual biological networks and their modulation through lifestyle, diet, and microbial engineering. Recent advances in microbiome-guided precision medicine highlight that the microbiota functions as a dynamic and programmable interface between host genetics and environmental inputs, mediating immunity, metabolism, neuroendocrine signaling, and pharmacological response (Shahid, 2025). Multi-omic platforms now provide the computational and analytical infrastructure necessary to decode these multidimensional interactions, linking microbial composition and function with host phenotypes at unprecedented resolution (Duan et al., 2024).

Multi-omic integration frameworks have advanced from descriptive association studies toward mechanistic modeling of host–microbe interactions. Integrative analyses combining metagenomics, metatranscriptomics, and metabolomics delineate how microbial metabolites act as systemic effectors that influence gene expression, immune tone, and metabolic homeostasis (Chetty and Blekhman, 2024; Muller et al., 2024). For example, network-based models and canonical correlation approaches identify cross-omic modules, coordinated shifts in microbial taxa, host pathways, and metabolites, that predict disease states or therapeutic outcomes. The MintTea algorithm exemplifies this new generation of integration tools by detecting disease associated multi-omic modules with high predictive power and biological coherence across cohorts (Muller et al., 2024). Such frameworks enable the design of biohacking interventions tailored to individual molecular signatures, allowing users to fine-tune circadian alignment, diet composition, and microbial modulation strategies according to their unique omic landscape.

Beyond mechanistic insight, multi-omics enables predictive modeling and risk stratification. The integration of polygenic risk scores (PRS) with gut metagenomic profiles has demonstrated improved predictive performance in research settings for complex diseases, including coronary artery disease, diabetes, Alzheimer’s, and prostate cancer, compared to conventional risk models (Muller et al., 2024). An integrated framework reveals that host genomic predisposition and microbial signatures interact to shape individual health trajectories. Within preventive biohacking, the fusion of such data can inform early lifestyle interventions, dietary supplementation, or probiotic strategies before pathological manifestations emerge. Longitudinal multi-omic analyses in healthy individuals also show that molecular subgroups maintain stable yet distinct metabolomic and lipidomic profiles predictive of future dysregulation (Kioroglou et al., 2025).

Personalization also extends to pharmacological domains. Pharmacomicrobiomics, a field merging pharmacogenomics with microbiome science, demonstrates that inter-individual variability in drug response is shaped not only by host genetics but also by microbial metabolism and signaling. Gut microbes modulate drug absorption, distribution, and metabolism, thereby altering therapeutic efficacy and toxicity profiles (Doestzada et al., 2018). Conversely, pharmaceuticals can remodel microbial communities, generating feedback loops that affect long-term metabolic and immune homeostasis. Multi-omic integration of host and microbial genomic data, coupled with metabolomic tracking of drug-derived compounds, provides a framework for precision dosing and probiotic co-therapies aimed at enhancing drug efficacy while minimizing adverse effects (Zhao et al., 2023; Gronich et al., 2025).

Recent advances in engineered biology and cyber-biological convergence further expand the frontier of precision biohacking. Engineered microbes can now be programmed to sense and modulate host biochemistry, supported by digital infrastructures for data acquisition and algorithmic feedback control. This cyber-bio integration blurs the boundary between biological and computational systems, allowing for the remote monitoring and modulation of host physiology. However, as biological systems exhibit self-assembly and self-repair capabilities, cyberbiosecurity becomes an essential component of responsible biohacking design (Elgabry and Johnson, 2024).

At the same time, the incorporation of probiotics and next-generation beneficial microbes into multi-omic pipelines refines personalization at the microbial level. Multi-omics facilitates the characterization and selection of probiotic strains based on their genomic, proteomic, and metabolomic profiles, ensuring compatibility with host physiology and disease context. This integrative view transcends the “one-strain-fits-all” paradigm, moving toward individualized microbial consortia capable of targeted metabolite production, immune modulation, and barrier reinforcement. For instance, species such as *Akkermansia muciniphila* and *Faecalibacterium prausnitzii* are being integrated into probiotic formulations designed through system-biology modeling to enhance metabolic resilience and anti-inflammatory capacity (Kwoji et al., 2023; Verma et al., 2025).

Temporal meta-omics studies provide yet another layer of personalization by elucidating how microbial ecosystems dynamically adapt to external perturbations. Longitudinal analyses integrating metagenomic, metatranscriptomic, proteomic, and metabolomic data have shown that microbial communities exhibit rapid functional plasticity and resilience following environmental or dietary disturbances. Adaptive capacity can be leveraged for precision interventions, where controlled perturbations, through fasting, nutrient cycling, or targeted microbial supplementation, reprogram ecosystem stability and enhance host resilience (Herold et al., 2020; Rozera et al., 2025).

### Mechanistic pathways linking microbial metabolites signalling and host-microbiome crosstalk

Extensive mechanistic evidence supports the role of microbial metabolites as mediators of host-microbiome communication, although causal relationships in humans remain context dependent and pathway-specific. Microbial metabolites act as central molecular mediators in the bidirectional communication between the host and its associated microbiota, integrating metabolic, immune, endocrine, and epigenetic pathways that shape physiological and pathological states. Among these metabolites, SCFAs such as acetate, propionate, and butyrate have been identified as key regulators of host-microbe signaling networks. These molecules are produced through microbial fermentation of dietary fibers by dominant taxa in the phyla *Firmicutes* and *Bacteroidetes*, reaching high concentrations in the proximal colon and serving as signaling ligands for host receptors including G protein-coupled receptors (GPR41/43) and free fatty acid receptors (FFAR2/3). Once absorbed, SCFAs modulate epithelial integrity by enhancing tight junction assembly and mucus production, exerting anti-inflammatory effects through inhibition of histone deacetylases (HDACs) and promotion of regulatory T cell (Treg) differentiation. Butyrate in particular acts as both an energy source for colonocytes and an epigenetic modulator, integrating microbial metabolic activity with host immune tolerance (Wang et al., 2024; Kim, 2021).

Beyond SCFAs, a diverse spectrum of microbial metabolites, including bile acid derivatives, polyamines, tryptophan catabolites, and itaconate, contribute to complex host signaling pathways. Microbiota-derived bile acids (BAs), through their interaction with nuclear receptors such as FXR and PXR, regulate intestinal immune homeostasis and metabolic processes, linking microbial metabolic activity to epithelial renewal and inflammation control. These BAs undergo microbial deconjugation and transformation into secondary bile acids, which in turn modulate inflammatory cascades, barrier defense, and tumorigenic signaling pathways (Cai et al., 2022; Fogelson et al., 2023). Tryptophan-derived indole catabolites further shape mucosal immunity through activation of the aryl hydrocarbon receptor (AhR), enhancing epithelial defense and suppressing pro-inflammatory Th17 activity. Itaconate, generated via the immune response gene 1 (IRG1) pathway in myeloid cells, mediates antibacterial activity while simultaneously controlling inflammatory signaling through inhibition of succinate dehydrogenase, thereby influencing the metabolic-immune interfaceV (Zhang et al., 2023).

A mechanistic dimension arises from the epigenome-microbiome axis, where microbial metabolites reshape host chromatin structure and modulate gene expression. Evidence shows that microbial short-chain fatty acids and other metabolites regulate histone acetylation, DNA methylation, and noncoding RNA activity in epithelial and immune cells, forming a molecular pathway through which microbiota-mediated environmental cues influence host physiology. In turn, host epigenetic reprogramming can modify gut microbial composition and function, establishing a feedback loop that integrates environmental factors, signaling pathways, and microbial ecology (Pepke et al., 2024).

Beyond microbial metabolites, insights from viral oncology highlight how biological systems can be epigenetically reprogrammed through external molecular signals. Oncogenic viruses such as HPV, EBV, HBV, HCV, and HTLV-1 induce host chromatin remodeling by altering DNA methylation, histone acetylation, and enhancer accessibility, leaving persistent epigenetic imprints that shape inflammation, immune evasion, and tumor progression (Bautista and Lopez-Cortes, 2025). This natural form of “viral biohacking” mirrors the mechanisms targeted in therapeutic and synthetic biology interventions. Pharmacologic DNMT and HDAC inhibitors, EZH2 blockade, and oncolytic virotherapy exemplify controlled strategies to reverse pathogenic silencing and restore antitumor immunity, illustrating the translational potential and ethical responsibility of harnessing programmable epigenetic modulation within precision biohacking frameworks (Hitch et al., 2022).

The crosstalk between microbial metabolites and host immunity involves multiple molecular pathways. SCFAs act through GPR43 and GPR109A signaling to modulate cytokine networks, increase IL-10 production, and support Treg stability, while suppressing NF-κB-dependent pro-inflammatory signaling (Takeuchi et al., 2024; Kim, 2021). Similarly, microbial bile acids regulate inflammasome activation and IL-18 production, thereby contributing to barrier defense and immune tolerance. Polyamines such as spermidine and putrescine, produced through microbial amino acid metabolism, modulate autophagy and cellular proliferation in mucosal tissues, supporting epithelial integrity (Zhang et al., 2023). Furthermore, tryptophan catabolites generated by *Lactobacillus* and other commensals activate AhR signaling, leading to the expression of IL-22 and antimicrobial peptides, critical components of mucosal defense (Losol et al., 2024; Cai et al., 2022).

Systemically, microbial metabolites have endocrine-like functions, influencing distant organs through circulation. For example, acetate modulates hepatic gluconeogenesis, while propionate affects adipose tissue metabolism, thereby integrating microbiota activity into host energy balance. Bile acid derivatives exert hormonal signaling via FXR-FGF15/19 axes, shaping liver metabolism and immune responses beyond the gut. Likewise, neuroactive metabolites such as GABA, serotonin precursors, and trimethylamine N-oxide (TMAO) have been implicated in gut-brain axis signaling, influencing neuroinflammation and behavior (Zhang et al., 2023; Fogelson et al., 2023; Ahmed et al., 2022).

In the context of immune regulation, microbial metabolites exert fine control over both innate and adaptive responses. SCFAs promote the expansion and functional specialization of regulatory T cells, support the development of tolerogenic dendritic cells, and influence B cell differentiation, promoting IgA production (Kim, 2021). Simultaneously, bile acid signaling shapes the composition of intraepithelial lymphocytes and innate lymphoid cell subsets, particularly ILC3, which play central roles in mucosal barrier protection. Immune interactions operate bidirectionally, as immune-derived signals such as IFNγ and IL-22 can modify microbial metabolic activity, revealing a reciprocal regulatory dynamic within the host-microbiome axis (Cai et al., 2022; Jimenez-Sanchez et al., 2025).

Mechanistically, this crosstalk operates through receptor-mediated signaling (e.g., GPCR, FXR, AhR), epigenetic modulation (HDAC inhibition, methylation patterns), metabolic reprogramming (e.g., TCA cycle rewiring), and immune-neuronal pathways (e.g., vagal signaling). Such integrated mechanisms form the molecular backbone of the holobiont framework, positioning the microbiome as an active participant in maintaining immune homeostasis and systemic metabolic health (Pepke et al., 2024; Ahmed et al., 2022). Dysregulation of these pathways, whether through dietary changes, antibiotic exposure, or disease, can disrupt metabolite signaling, leading to chronic inflammation, impaired barrier function, and increased susceptibility to diseases such as IBD, cancer, metabolic syndrome, and neurodegeneration (Ullah et al., 2024; Cai et al., 2022).

### Microbiome-gut-brain axis in cognitive enhancement mood regulation and neurohacking

The microbiome-gut-brain axis (MGBA) represents a complex bidirectional communication network that integrates intestinal microbial communities with the central nervous system (CNS) through neural, endocrine, immune, and metabolic signaling pathways. Growing evidence shows that microbiota-derived metabolites, neurotransmitters, and immune mediators modulate cognition, emotion, and neural plasticity, forming a biological basis for both clinically oriented microbiome-brain research and emerging neurohacking practices (Ienca and Scheibner, 2020; Xu and Lu, 2025).

Mechanistically, the MGBA functions through intertwined pathways involving the vagus nerve, the hypothalamic–pituitary–adrenal (HPA) axis, and the gut-immune interface. These systems translate microbial activity into neurochemical outputs such as serotonin (5-HT), dopamine, *γ*-aminobutyric acid (GABA), and SCFAs, which regulate mood, memory, and executive function (Loh et al., 2024). Microbial dysbiosis has been linked to neuropsychiatric and neurodegenerative disorders, while restoration of eubiosis through psychobiotics, prebiotics, and synbiotics has consistently improved depression and anxiety outcomes in clinical trials and meta-analyses (Radjabzadeh et al., 2022; Pan et al., 2025).

From a psychobiological perspective, psychobiotics, live microorganisms that confer mental health benefits, modulate the MGBA by balancing cytokine profiles, restoring excitatory-inhibitory neurotransmission, and reducing HPA axis hyperactivation (Kamal et al., 2025). Specific *Lactobacillus* and *Bifidobacterium* strains enhance tryptophan metabolism and SCFA synthesis, promoting serotonergic tone and brain-derived neurotrophic factor (BDNF) signaling. Conversely, depletion of anti-inflammatory genera such as *Faecalibacterium* or *Coprococcus* correlates with increased depressive symptoms and disrupted GABA metabolism. A large microbiome-wide association study identified 13 bacterial genera, including *Eggerthella*, *Sellimonas*, *Subdoligranulum*, and *Lachnoclostridium*, whose altered abundance correlates with depression via impaired production of glutamate, serotonin, and GABA. Complementary neuroimmunological models emphasize the gut-immune-brain axis, where microbial antigens modulate systemic cytokine responses and microglial activation, shaping the inflammatory tone of the CNS (O’Riordan et al., 2025; Park et al., 2025).

Recent conceptual advances incorporate circadian rhythmicity as a key temporal dimension of the MGBA. Microbial and host endocrine networks exhibit synchronized diurnal oscillations that align immune, metabolic, and neurotransmitter activity. Disruption of this synchrony, through irregular feeding, sleep deprivation, or chronic stress, dampens SCFA and tryptophan metabolite rhythms, destabilizes HPA axis regulation, and promotes neuroinflammation, increasing vulnerability to anxiety and cognitive decline. Through microbiota-clock crosstalk involving *CLOCK*, *BMAL1*, *PER*, and *CRY* genes, gut-derived signals influence cortisol release, cytokine tone, and vagal activity, establishing a gut-brain-circadian axis that sustains mood and cognitive stability. Chronobiological misalignment thus represents a time-dependent form of dysbiosis, while chrononutrition, time-restricted feeding, and circadian-timed psychobiotics emerge as novel neurohacking strategies to restore rhythmic microbial-neural coherence (Bautista and López-Cortés, 2025a; Bautista et al., 2025h; Rusch et al., 2023).

The vagus nerve serves as the principal conduit for gut to brain signaling and plays a central role in emotional regulation. It detects microbial metabolites and relays them to limbic and cortical centers governing emotion, motivation, and cognition. Experimental vagus nerve stimulation (VNS) enhances monoaminergic transmission and synaptic plasticity, alleviates treatment-resistant depression and epilepsy, and concurrently normalizes gut microbial composition, demonstrating a bidirectional microbiota-vagal feedback loop essential for emotional and cognitive homeostasis. Moreover, specialized intestinal neuropods that sense bacterial metabolites underscore the rapid electrochemical transduction linking gut microbes to higher neural circuits (Faraji et al., 2025).

Beyond mood regulation, MGBA-mediated neurometabolic crosstalk drives cognitive enhancement strategies within neurohacking. This emerging field encompasses interventions, ranging from dietary modulation, nootropics, and probiotics to bioelectronic interfaces, designed to explore the modulation of cognitive capacity and emotional resilience. Ethical neurohacking integrates microbial engineering and multi-omics monitoring to fine-tune neurotransmitter biosynthesis, inflammation, and synaptic efficiency. SCFAs produced by *Butyrivibrio* and *Roseburia* act as histone deacetylase inhibitors, upregulating neuronal gene expression linked to learning and memory (Moshfeghinia et al., 2025; Loh et al., 2024) Similarly, microbial metabolites derived from tryptophan catabolism regulate the kynurenine pathway and aryl hydrocarbon receptor signaling, modulating neurogenesis and resistance to stress-related mood disorders (O’Riordan et al., 2025).

At the neuroimmune interface, the gut microbiota modulates innate and adaptive immunity, influencing both neuroinflammation and cognition. Butyrate reinforces the blood–brain barrier, regulates microglial maturation, and preserves synaptic integrity under stress or aging. Conversely, impaired gut-immune communication fosters chronic low-grade inflammation and defective clearance of neurotoxic aggregates, contributing to neurodegeneration (Moshfeghinia et al., 2025). Integrating microbiota-targeted interventions, such as diet-induced eubiosis, engineered probiotics, or FMT, with immunomodulatory strategies holds translational promise for improving cognitive flexibility and emotional stability (Pan et al., 2025).

Meta-analyses confirm the efficacy of microbiome-based interventions, reporting significant reductions in depression and anxiety symptoms, with standardized mean differences from −0.26 to −1.76 across populations. Variations in therapeutic response reflect the influence of diet, genetics, and baseline microbial composition (Radjabzadeh et al., 2022; Pan et al., 2025). Neuroimaging and metabolomic studies further associate psychobiotic intake with enhanced prefrontal-limbic connectivity and elevated circulating SCFAs and serotonin precursors (Park et al., 2025). In viral-associated neurodegeneration, such as herpes simplex virus-1 (HSV-1) infection, gut dysbiosis intensifies neuroinflammation, whereas microbial restoration mitigates microglial activation and maintains proteostasis, suggesting microbiota-dependent regulation of neuroimmune defense (Bautista et al., 2025a).

Ultimately, the convergence of microbiome science and neurotechnology redefines cognitive enhancement within an ethical and biological continuum. Multi-omics platforms linking metagenomics, metabolomics, and neuroimaging enable individualized mapping of gut-derived neurotransmitter and immune networks (O’Riordan et al., 2025). Expanding this concept, recent evidence integrates circadian and neurodegenerative signaling into the MGBA, revealing that microbial and host oscillations coordinate endocrine and immune rhythms essential for cognitive stability. Disruption of *CLOCK* and *BMAL1* expression desynchronizes HPA axis function and amplifies neuroinflammation, whereas chrononutritional and microbiota-targeted interventions restore rhythmic signaling and neuroplasticity, offering the foundation for temporal neurohacking (Bautista et al., 2025f).

Moreover, neuro-immune interactions indicate that neurodegeneration and dysbiosis share convergent signaling networks affecting cognition and stress resilience. Degenerating neurons and reactive glia release cytokines and metabolites that alter gut microbial composition, establishing a neuro-immune-microbiota feedback loop that sustains chronic inflammation and synaptic remodeling. Central regulators such as p53, STING, and NF-κB integrate oxidative stress, immune activation, and microbial metabolism, linking neuronal injury to systemic inflammation and barrier dysfunction (Bautista et al., 2025c; Park et al., 2025; Fan et al., 2025).

### Metabolic biohacking in obesity insulin sensitivity and longevity

Metabolic biohacking can be understood as the deliberate, often self-directed manipulation of lifestyle, diet, microbiome and pharmacology to optimize metabolic control, body composition and healthy lifespan. In diabetes and obesity, biohacks range from extreme low-carbohydrate or intermittent fasting regimens to DIY use of wearables, nutraceuticals and unregulated “anti-aging” compounds, frequently implemented outside formal medical supervision. While such practices may empower individuals and promote engagement with health, they also blur the boundary between evidence-based care and non-validated experimentation. Therefore, “metabolic biohacking” should be re-framed as the rational, data-informed use of validated interventions, particularly those targeting the gut microbiome, to enhance insulin sensitivity, reduce obesity-related risk and support healthy aging, while explicitly recognizing the ethical and sociocultural implications of enhancement and life-extension discourses (Kalra et al., 2025; Cozza et al., 2022; Kadyan et al., 2025). Throughout this section, metabolic biohacking is discussed within a framework that prioritizes evidence-based interventions and explicitly distinguishes them from non-validated or speculative practices.

The gut microbiome has emerged as a central target for metabolic biohacking because it integrates dietary signals, immune tone and energy harvest, all of which shape obesity and insulin resistance trajectories (Mederle et al., 2024). Dysbiosis, typically characterized by reduced diversity, shifts in the Firmicutes/Bacteroidetes ratio, expansion of pathobionts and loss of key butyrate-producing taxa, has been consistently linked to increased appetite, low-grade inflammation, visceral adiposity and impaired insulin signaling in obesity, metabolic syndrome and type 2 diabetes. Mechanistically, the microbiome modulates intestinal barrier integrity, bile acid pools, SCFA production and gut-derived hormones, thereby influencing both systemic insulin sensitivity and cardiometabolic risk, key determinants of healthspan and lifespan (Crudele et al., 2023; Sasidharan Pillai et al., 2024; Kadyan et al., 2025).

Microbiome-targeted interventions represent prototypical evidence-based metabolic biohacks. A recent systematic review and meta-analysis of randomized trials showed that probiotics, prebiotics, synbiotics, FMT and diet-based strategies significantly reduce fasting glucose, HbA1c, HOMA-IR and atherogenic lipid fractions, while modestly increasing HDL-cholesterol in individuals with obesity, diabetes or metabolic syndrome. Narrative syntheses of obesity interventions similarly highlight the potential of high-fibre, Mediterranean-style and, in selected cases, ketogenic diets, alongside probiotics, FMT and structured physical activity, to reprogram gut microbial composition, increase SCFA production and facilitate weight loss and metabolic control (Mederle et al., 2024; Yarahmadi et al., 2024; Bautista et al., 2026d). Long-term data from adolescents with obesity indicate that a single course of encapsulated FMT can induce durable shifts in microbiome richness and function, with sustained improvements in waist circumference, total body fat, HDL-cholesterol and markers of systemic inflammation, even in the absence of major BMI changes (Wilson et al., 2025).

At the mechanistic level, multi-omics studies have begun to dissect how specific microbial pathways can be leveraged, or inadvertently perturbed, by metabolic biohacks. A large integrative analysis showed that individuals with insulin resistance harbor increased faecal monosaccharides and SCFAs, particularly propionate, linked to microbial carbohydrate metabolism and pro-inflammatory cytokine signatures (Takeuchi et al., 2023). These data suggest that excessive microbial breakdown of complex carbohydrates may fuel hepatic gluconeogenesis and systemic insulin resistance, providing a rationale for targeted dietary modulation of fermentable substrates and selective promotion of insulin-sensitizing taxa. In parallel, experimental and clinical evidence indicates that butyrate-producing bacteria and their metabolites enhance gut barrier function, regulate appetite and improve insulin signaling, whereas an overrepresentation of acetate-associated pathways may promote adiposity. Pharmacologic agents commonly incorporated into metabolic biohacking, such as GLP-1 receptor agonists, orlistat and semaglutide, also interact with the microbiome, indirectly reshaping bile acids, energy harvest and inflammatory tone, which may amplify or constrain their clinical benefits (Yarahmadi et al., 2024; Crudele et al., 2023).

Insulin sensitivity is a particularly attractive endpoint for microbiome-informed biohacking because it integrates diet, adiposity and inflammatory status and predicts cardiometabolic and longevity outcomes. Bibliometric analyses reveal a rapidly expanding research landscape dissecting the gut microbiota-insulin resistance axis, with convergent themes around high-fat diet, endotoxemia, intestinal permeability and inflammatory signaling as drivers of impaired insulin action (Zyoud et al., 2025). Multi-omics work links specific carbohydrate-metabolizing bacteria and their metabolites to HOMA-IR, metabolic syndrome and prediabetes, while identifying insulin-sensitivity-associated taxa capable of ameliorating insulin resistance in experimental models (Takeuchi et al., 2023; Crudele et al., 2023; Sasidharan Pillai et al., 2024). Within this framework, metabolic biohacking can be conceptualized as the personalized manipulation of diet, timing of food intake, microbiome-directed supplements and physical activity to favor insulin-sensitizing microbial networks, reduce gut-driven inflammation and preserve *β*-cell function across the lifespan (Mederle et al., 2024; Yarahmadi et al., 2024).

Finally, the links between microbiome, inflammaging and age-related disease position microbiome-centered biohacking as a promising, yet double-edged, approach to promote longevity. Age-associated “biome-aging” encompasses progressive loss of microbial diversity, expansion of pathobionts and chronic low-grade inflammation, all of which contribute to frailty, cardiometabolic disease and neurodegeneration. Emerging microbiome-based therapeutics, including next-generation probiotics, prebiotics, postbiotics, personalized nutrition and rational FMT, aim to counteract biome-aging, extend healthspan and possibly lifespan, but they also raise questions about equity, safety and the normalization of technologically mediated “enhancement” (Kadyan et al., 2025; Cozza et al., 2022).

### Risks, safety, regulatory and ethical considerations of microbiome biohacking

The rapid growth of microbiome biohacking, ranging from DIY biology, self-experimentation, consumer probiotics, and direct microbiome modulation, has generated substantial scientific enthusiasm but also a complex landscape of risks and ethical challenges. Activities performed outside controlled laboratory settings often bypass rigorous methodological, safety and ethical safeguards, increasing uncertainty about unintended personal and societal consequences. As highlighted by the expanding DIY biohacker movement, the lack of oversight, quality control, and standardized procedures can transform self-directed microbiome interventions into emerging public health risks. Efforts to democratize biological experimentation must therefore be matched with stringent risk communication, awareness of potential harms, and frameworks that protect both individuals and communities (Gaspar et al., 2019; Garg et al., 2024; Merenstein et al., 2023).

A central concern is scientific robustness and reproducibility, which represents a major vulnerability when microbiome manipulation is attempted without standardized protocols. Microbiome science requires careful sampling, sequencing, bioinformatics, and contextual interpretation, yet many personal biohacking practices rely on oversimplified or unvalidated assumptions about gut “balance” or “dysbiosis.” The STORMS guidelines stress the necessity of rigorous methodological reporting to ensure reproducibility and avoid overinflated causal claims, issues frequently observed in both commercial microbiome testing and amateur biohacking (Mirzayi et al., 2021; Lange et al., 2022).

Safety considerations remain paramount, particularly regarding probiotics, next-generation live biotherapeutics, and FMT. Although probiotics are generally recognized as safe, emerging evidence demonstrates that strain-specific properties, horizontal gene transfer, virulence factors, and antimicrobial resistance require deeper evaluation, particularly in vulnerable populations (Rodriguez et al., 2025). Case-by-case genomic characterization, including whole-genome sequencing, is recommended to assess safety, especially as users increasingly self-administer unregulated products or experimental strains. FMT exemplifies the tension between therapeutic promise and safety: despite its clinical potential, unregulated or home-based FMT poses serious risks of pathogen transmission, adverse immune reactions, and unpredictable ecological shifts in the host microbiome (Thanush et al., 2023; McGuinness et al., 2024).

Regulatory heterogeneity adds another layer of complexity. Probiotics, live biotherapeutics and microbiome therapies fall under diverse regulatory categories depending on jurisdiction, ranging from foods and dietary supplements to medicinal products, resulting in inconsistent standards and consumer protections. Regions such as Europe apply more stringent requirements, often limiting health claims and demanding robust clinical evidence, while other regions allow broader claims, potentially increasing exposure to poorly substantiated or misleading information. Recently, regulatory science initiatives have emerged to address the unique challenges posed by microbiome-based therapies, including quality specification, donor screening, clinical trial design, and long-term ecological monitoring (Thanush et al., 2023; Garg et al., 2024).

Beyond safety and regulation, ethical considerations represent a foundational pillar in evaluating microbiome biohacking. Ethical analyses emphasize principles of non-maleficence, autonomy, justice, and respect for all forms of microbiome-associated life. Personal autonomy in self-experimentation must be balanced against risks of misinformation, unequal access to safe interventions, and potential harm to individuals or ecosystems. Additionally, microbiome data itself raises questions of privacy, ownership, and equitable reuse (Lange et al., 2022; Merenstein et al., 2023; Hardwick et al., 2024). The recent call for equitable data-sharing practices and the implementation of Data Reuse Information (DRI) tags highlights the need to recognize data creators while preventing misuse or exploitation, a critical issue as microbiome sequencing becomes increasingly common among citizen scientists (Hug et al., 2025).

From a societal perspective, engineering human or environment-associated microbiomes carries implications for justice, equity, and public trust. Precision microbiome interventions may unintentionally reinforce structural inequities if their risks and benefits are not distributed fairly, particularly in marginalized communities historically overexposed to environmental and health risks. Ethical frameworks urge anticipatory governance, transparent communication, and participatory decision-making to ensure microbiome innovation evolves responsibly (Lange et al., 2022).

### Methodological challenges, reproducibility and standardization in microbiome biohacking research

The rapid expansion of microbiome biohacking has outpaced the methodological rigor traditionally expected in biomedical sciences, creating significant challenges in data generation, interpretation, and reproducibility. A central limitation lies in the heterogeneity of sampling strategies, sequencing platforms, and analytical pipelines used to characterize microbial communities in human subjects. Variations in sample collection timing, preservation media, storage temperature, and DNA extraction methods introduce substantial biases in microbial abundance and diversity estimates (Sandberg and Nelson, 2020; Bharti and Grimm, 2021). Evidence from clinical microbiome interventions, particularly perinatal and maternal studies, further demonstrates that inconsistent protocols, unvalidated dosing, and heterogeneous analytical standards undermine reproducibility and safety assessment. Integrating multi-omic and systems-biology frameworks has therefore been proposed to harmonize data generation and improve predictive accuracy, enabling ethically governed and clinically translatable applications (Zack et al., 2025; Mills et al., 2025; Bautista and López-Cortés, 2025c). Pre-analytical discrepancies collectively impair cross-study comparability and limit the development of robust biomarkers for personalized biohacking interventions. Moreover, the proliferation of commercial at-home testing kits, often lacking standardized methodologies, exacerbates variability and restricts the generalizability of findings beyond niche consumer populations (Bokulich and Robeson, 2024; Panek et al., 2018).

Sequencing methodologies present an additional layer of complexity. The choice between 16S rRNA gene profiling, metagenomics, metatranscriptomics, or multi-omics approaches dramatically influences the taxonomic and functional resolution achieved. While 16S remains widely used due to its affordability and accessibility, it suffers from primer bias, limited taxonomic depth, and susceptibility to false positives from contaminants (Matchado et al., 2024). Shotgun metagenomics improves strain-level identification and gene-content profiling but requires higher sequencing depth, stringent quality control pipelines, and advanced computational infrastructure, creating disparities in data reliability and analytical reproducibility between academic laboratories and community biohacking groups (Panek et al., 2018; Liu et al., 2022). The absence of consensus-driven best practices for quality filtering, read assembly, and functional annotation further contributes to inconsistent outputs, impairing reproducibility and making cross-study comparisons challenging (Fierer et al., 2025; Yu et al., 2024).

Bioinformatic variability constitutes one of the most critical but under-recognized methodological bottlenecks in microbiome biohacking research. Different taxonomic classifiers, reference databases, and statistical normalization approaches can produce divergent interpretations from identical datasets (Bokulich and Robeson, 2024). For example, the use of compositional data methods versus traditional relative abundance metrics yields markedly different associations between microbial taxa and host phenotypes, directly influencing conclusions about which interventions, dietary, probiotic, or metabolic, are effective (Bharti and Grimm, 2021; Yu et al., 2024). Additionally, many biohacking studies rely on correlation-based analyses without incorporating longitudinal designs or causal inference frameworks, leading to oversimplified assumptions regarding microbial causality. The methodological gap restricts the ability to distinguish transient, noise-driven fluctuations from biologically meaningful shifts induced by interventions (Austin and Korem, 2024; Novak et al., 2025).

Standardization of intervention protocols remains another substantial challenge. Biohacking practices often include personalized diets, probiotics, fasting strategies, metabolite supplementation, or FMT. However, these interventions are rarely administered with consistent dosing, duration, or monitoring procedures across studies or self-experimenters. Variability in probiotic strain viability, formulation stability, and host colonization capacity further complicates interpretation, as many commercially available products are not rigorously validated to replicate the effects observed in controlled trials. Similarly, the integration of wearable devices, digital biomarkers, or self-reported metrics into microbiome-focused protocols introduces noise related to device calibration, user compliance, and subjective reporting (Fierer et al., 2025; Williamson et al., 2025; Hertel et al., 2023; Bautista et al., 2026c).

Ethical and regulatory considerations intersect with methodological limitations. Data derived from unsupervised experimentation and consumer-level sequencing platforms may lack crucial metadata, including diet, medication, circadian rhythms, and lifestyle habits, all key determinants of microbial composition (Austin and Korem, 2024; Drago, 2025). Without robust metadata standards, attempts to replicate findings or predict responses to microbiome-targeted interventions become inherently flawed. The problem becomes more pronounced within open-science and citizen-science biohacking communities, where datasets frequently lack proper controls, defined clinical endpoints, and standardized consent procedures (Williamson et al., 2025; Yu et al., 2024; Metris et al., 2025).

Reproducibility issues are compounded by the dynamic and context-dependent nature of the human microbiome. Inter-individual variability is influenced by genetics, environmental exposures, age, hormonal status, geography, and long-term dietary patterns, factors that are rarely controlled in biohacking studies (Bokulich and Robeson, 2024). Even within the same individual, intra-day and intra-week fluctuations occur due to sleep, stress, meal timing, and physical activity (Bharti and Grimm, 2021). Without high-resolution longitudinal sampling and validated normalization pipelines, distinguishing intervention-driven effects from natural variability remains nearly impossible. Thus, methodological standardization must move beyond static guidelines and incorporate temporal, ecological, and personalized dimensions of microbial dynamics (Fierer et al., 2025; Silverman et al., 2018).

To address these challenges, the field increasingly recognizes the need for harmonized frameworks that integrate standardized sample processing, sequencing, statistical modeling, and reporting guidelines. Initiatives advocating for universal metadata templates, reference standards, mock microbial communities, and validated computational pipelines offer promising strategies to enhance reproducibility (Metris et al., 2025; Keenum et al., 2025). Moreover, multi-omics integration, combining metagenomics, metatranscriptomics, metabolomics, and host immunomics, provides a more biologically coherent characterization of host–microbe interactions, but requires rigorously reproducible pipelines to avoid compounding methodological noise. Transparent reporting of intervention protocols, strain-level validation for probiotics, and open-source documentation of bioinformatic workflows are critical steps for bridging the gap between experimental research and real-world biohacking applications (Boyte et al., 2023; Williamson et al., 2025). Achieving methodological rigor and transparency will be pivotal to transform microbiome biohacking from anecdotal experimentation into a reproducible scientific discipline.

## Conclusions and future perspectives

The convergence of biohacking, microbiome science, and precision medicine defines an emerging and potentially transformative frontier in human health optimization. Emerging evidence demonstrates that targeted manipulation of the human microbiome, combined with real-time physiological feedback and multi-omic monitoring, enables a new level of metabolic, cognitive, and immunological control that transcends traditional medical paradigms. Biohacking techniques, from nutrigenomics and intermittent fasting to engineered microbial consortia and wearable sensors, recalibrate host-microbe networks, fine-tune energy metabolism, and modulate immune resilience. Interventions of this kind mark a shift from reactive disease management to proactive self-regulation, where individuals intentionally modulate their internal ecosystems to maintain health and extend longevity. Microbial metabolites such as SCFAs, polyamines, and tryptophan derivatives act as biochemical mediators linking diet, microbiota, and host physiology, underscoring the microbiome’s role as a programmable endocrine and immunometabolic interface (Jairoun, 2025; Gangadharbatla, 2020; Zhang et al., 2023).

Beyond metabolic modulation, biohacking integrates the digital and biological realms, expanding human-machine symbiosis. The transition from wearables to embedded interfaces, biostamps, subdermal chips, and biosensing implants, allows continuous bidirectional communication between body and device, converting physiological data into actionable insights (Kalra et al., 2025). Such embedded technologies extend the quantified-self movement toward a cyber-biological continuum, where health is monitored and adjusted through predictive algorithms. However, these same advances raise critical concerns regarding data privacy, algorithmic bias, and bodily autonomy. The integration of microbiome-derived data into networked digital infrastructures necessitates robust cybersecurity and ethical governance frameworks. As engineered biology becomes increasingly automated and cloud-linked, vulnerabilities such as synthetic-sequence piracy and biological dataset targeting demand the creation of adaptive cyberbiosecurity systems that safeguard both genomic and microbial information (Elgabry and Johnson, 2024).

Parallel developments in neurohacking reveal how direct modulation of brain activity can intersect with microbial and metabolic control to optimize cognition, emotion, and behavior. Through brain-computer interfaces, neuromodulation, and cognitive feedback systems, neurohacking aims to enhance attention, memory, and resilience, yet also blurs ethical boundaries between therapy and enhancement. The microbiome-gut-brain axis, in which microbial metabolites influence neurotransmitter synthesis and neuroplasticity, provides a biological substrate linking these domains, illustrating how microbial engineering could interface with neurotechnology for cognitive health. Ethical and legal frameworks must therefore evolve to ensure informed consent, transparency, and protection against misuse in self-experimentation and neuroenhancement (Ienca and Scheibner, 2020; Gaspar et al., 2019).

At a broader philosophical level, biohacking aligns with transhumanist ideals of overcoming biological constraints, particularly aging. The concept of “hacking age” reframes senescence as a reversible condition amenable to molecular, microbial, and technological intervention. By merging longevity science, nutrigenomics, and microbiome modulation, biohacking aspires to extend healthspan while maintaining performance and vitality. Yet, this pursuit also exposes tensions between empowerment and the commodification of health, as enhancement practices risk reinforcing societal hierarchies tied to access, gender, and socioeconomic privilege (Song and Shin, 2022). In contrast, the democratization of biotechnology through citizen-science and DIY-bio movements fosters inclusivity and innovation but introduces new biosafety and ethical challenges. Responsible governance must balance open scientific participation with regulatory oversight, ensuring that biohacking remains a tool for public empowerment rather than a source of inequity or harm (Gaspar et al., 2019; Gangadharbatla, 2020).

Clinically, biohacking principles are already being integrated into chronic-disease management. In diabetes care, self-developed tools such as DIY artificial-pancreas systems exemplify patient-driven innovation, enhancing glucose control and self-efficacy while revealing the risks of unregulated self-experimentation (Cozza et al., 2022). In obesity medicine, biohacking-inspired interventions, combining fasting, nutrigenomics, and microbiome modulation, demonstrate measurable benefits in metabolic flexibility and weight regulation (Jairoun, 2025; Bautista et al., 2025b). Examples of this nature underscore the promise of microbiome innovation while emphasizing the need for structured ethical, clinical, and data-integrity standards to ensure safe translation into mainstream healthcare.

A crucial dimension of future biohacking lies in personalized nutrition guided by microbiome profiling. The interplay between dietary components, gut microbial composition, and host metabolic phenotype supports the design of individualized dietary regimens that transcend the “one-size-fits-all” approach. Integration of metabolomic and genomic readouts can optimize nutrient absorption, regulate immune tolerance, and prevent chronic diseases through precision dietary algorithms. Such microbiome-based personalization embodies the central tenet of biohacking, real-time adaptation of biological systems for optimized health trajectories, yet demands large-scale validation and harmonized bioinformatics standards to ensure reproducibility and equity of access (Bokulich and Robeson, 2024; Zhang et al., 2023; Bautista et al., 2026b).

Recent microbiome research has increasingly been discussed within the One Health framework, which conceptualizes human, animal, and environmental health as interconnected biological systems. From this perspective, microbial communities extend beyond individual hosts and participate in ecological networks that include environmental reservoirs and animal-associated microbiota. Concepts such as microbiome medicine and microbiome biohacking, which aim to modulate host-associated microbial communities, may therefore benefit from being interpreted within this broader ecological context. Integrating One Health perspectives may help situate human microbiome variation within the wider environmental and interspecies exchanges that shape microbial ecosystems (Muhummed et al., 2025; Zhou et al., 2024; Bautista et al., 2026a).

Looking forward, the next decade will see the convergence of AI-driven analytics, microbial engineering, and embedded biosensing converge into dynamic feedback ecosystems capable of continuously adjusting human biology. These “closed-loop” systems could monitor and recalibrate microbiome composition, immune markers, and metabolic outputs, moving health management from episodic to continuous and predictive. Achieving this vision will require integrating cyberbiosecurity, ethical oversight, and open scientific collaboration to prevent misuse and ensure transparency. The future of biohacking the human microbiome will thus depend not only on technological innovation but on cultivating a holistic framework that unites biology, ethics, and digital governance. In doing so, the field can transition from an experimental subculture to a scientifically grounded pillar of precision health, positioning the microbiome as a central interface for future therapeutic innovation within ethically governed precision health frameworks.
